# Regional disparities, dynamic evolution, and spatial spillover effects of medical resource allocation efficiency in TCM hospitals

**DOI:** 10.1186/s12962-025-00644-6

**Published:** 2025-07-18

**Authors:** Zhihao Wang, Zhiguang Li, Ruijin Xie

**Affiliations:** 1 The Second Affiliated Hospital of Anhui University of Chinese Medicine, Hefei, 230012 Anhui China; 2 School of Economics and Management, Anhui University of Chinese Medicine, Hefei, 230012 Anhui China; 3 Key Laboratory of Data Science & Innovative Development of Traditional Chinese Medicine, Philosophy and Social Sciences of Anhui Province, Hefei, 230012 Anhui China

**Keywords:** Traditional Chinese Medicine (TCM) hospitals, Medical and health resources, Allocation efficiency

## Abstract

**Background:**

To analyze the regional disparities, dynamic evolution, and influencing factors of medical resource allocation efficiency in TCM hospitals across China from 2016 to 2022, providing references for optimizing resource allocation in TCM hospitals.

**Methods:**

The study employed a super-efficiency Slack-Based Measure (SBM) model considering undesirable outputs to assess regional equity in efficiency, utilized the Dagum Gini coefficient to measure regional disparities in efficiency, and applied kernel density estimation and spatial econometric models to analyze the dynamic evolution and spatial spillover effects of medical resource allocation efficiency in TCM hospitals.

**Results:**

In 17 provinces, the efficiency is higher than the average value of 0.839, and in 8 provinces, the average value has exceeded 1. The regional pattern of efficiency shows a gradient characteristic of "high in the east and stable in the west, with the Northeast lagging behind." There is a significant spatial difference in the efficiency of resource allocation. The overall difference in the allocation of resources for traditional Chinese medicine (TCM) hospitals shows a fluctuating upward trend. The contribution rate of regional differences reaches 53.45%, which is the dominant factor. The largest regional differences are found within the central region, while the gaps between the eastern and central regions continue to widen, and those between the western and northeastern regions tend to become more balanced. The most significant interregional differences are observed between the central and western regions. The efficiency of resource allocation for TCM hospitals is on the rise, with the kernel density curve shifting to the right. The main peak height first decreases and then increases, while the width first expands and then contracts. The absolute difference first increases and then decreases. The rightward convergence of the tail indicates that there are efficient hospitals, but the gaps are narrowing. The multi-peak distribution reveals a multi-level differentiation pattern with the coexistence of low-efficiency and high-efficiency clusters. Per capita GDP, urbanization level, aging rate, population density, and the number of graduates from higher medical colleges can promote efficiency improvement. Population density and the proportion of TCM physicians have a positive spatial spillover effect on efficiency, while per capita GDP has a negative spatial spillover effect.

**Conclusion:**

The efficiency of medical resource allocation in traditional Chinese medicine (TCM) hospitals is steadily improving, and the regional differences are continuously narrowing. The degree of efficiency multi-polarization is becoming more moderate, and the development of regional equilibrium is being achieved. Both internal and external environmental factors jointly influence the improvement of medical resource allocation efficiency in TCM hospitals. It is recommended to take measures such as technological empowerment, institutional constraints, financial support, and talent absorption to enhance the efficiency of medical resource allocation in TCM hospitals and bridge the regional gaps.

## Introduction

Traditional Chinese medicine (TCM) hospitals serve as essential carriers of TCM service provision, playing a critical role in improving population health and advancing the Healthy China Initiative. In 2019, the *Opinions of the CPC Central Committee and the State Council on Promoting the Inheritance and Innovative Development of Traditional Chinese Medicine* outlined six major tasks, including strengthening the TCM service system, enhancing the role of TCM in disease prevention and treatment, and improving TCM workforce development. This policy document has become a strategic blueprint for TCM reform and development in China [1]. Complementing this, the *Strategic Plan for the Development of Traditional Chinese Medicine (2016–2030)* defines long-term goals for the sector, aiming to achieve full coverage of TCM services by 2030 [2].

Within this policy context, the equitable distribution of medical resources in TCM hospitals has emerged as a critical issue. It is not only an important objective in achieving sustainable development goals, but also a key governmental effort to improve people’s livelihoods and promote social equity [3–7]. The 20th National Congress of the Communist Party of China further emphasized the need to expand the supply of high-quality TCM services and optimize their regional distribution [5, 7]. In response, the government has promoted coordinated mechanisms to integrate high-quality healthcare resources and has established, to a certain extent, a hierarchical medical system aligned with China’s national conditions, helping to ease longstanding issues such as poor access to care and high treatment costs [4, 8].

Nevertheless, challenges persist. In reality, TCM healthcare resources remain predominantly concentrated in economically developed areas and urban centers, while access in rural and remote regions continues to be limited. This imbalance contributes to persistent urban–rural disparities in medical resource allocation [3, 5, 7]. To address this issue, it is imperative to develop methods for quantitatively assessing the urban–rural differences in healthcare demand under the hierarchical medical system. In addition, it is necessary to determine the optimal spatial locations for new primary, secondary, and tertiary TCM facilities and to construct a resource allocation model that simultaneously considers equity of service coverage, efficiency of resource use, and operational cost constraints. Such an approach would contribute to improving overall allocation efficiency, reducing regional disparities, and identifying the underlying drivers and spatial spillover effects of TCM healthcare resources—offering valuable insights for achieving coordinated regional development in TCM services [7].

In recent years, academic research on healthcare resource allocation efficiency has advanced significantly in terms of methodological frameworks, spatial scale, and research depth, resulting in a multi-dimensional and evolving research paradigm. From a methodological standpoint, early studies relied on traditional data envelopment analysis (DEA) for measuring single-dimensional efficiency [9, 10]. Later, the subsequent development of three-stage and four stage DEA models enables researchers to control the impact of external environment [11, 12]. Subsequently, the incorporation of fairness indicators such as the Gini coefficient and rank-sum ratio methods enriched the assessment of equity in healthcare distribution [13, 14]. More recently, the integration of spatial econometric models has allowed for the examination of spatial dependence and spillover effects, further enhancing the ecological validity and explanatory power of allocation studies [3, 7, 15].

In terms of spatial scale, research has progressed from macro to micro levels. At the global scale, studies have explored equity dynamics in cross-national healthcare resource allocation [16–18]. National-scale analyses have focused on the impacts of policy interventions on distribution mechanisms [19, 20]. At the regional level, attention has turned to spatial heterogeneity and inter-provincial coordination [21–23]. Finally, micro-level studies at the community scale have applied refined accessibility models to identify underserved areas and service gaps at the grassroots level [24]. This multi-tiered spatial assessment framework has significantly improved the precision and applicability of research findings. With respect to research depth, the field has evolved from initial descriptive analyses of spatial distribution patterns [25] to the identification of socioeconomic determinants and their influence pathways [26], and more recently to investigations of the coupling effects between institutional policies and market forces [27].

Building upon this body of work, this study proposes three contributions: Firstly, a novel Undesirable-Super-SBM model is constructed to address bias in traditional DEA models resulting from radial measurement and slack variables. By incorporating undesirable outputs and employing non-radial optimization, this model improves the accuracy of efficiency assessments in the context of TCM hospital resource allocation. Secondly, this study integrates the theory of unbalanced regional development from regional economics [28] with the theory of spatial equity from public health [29], thereby establishing an interdisciplinary analytical framework that can be applied to complex resource distribution scenarios. Thirdly, by exploring the influencing factors and spatial spillover effects that drive TCM resource allocation, the study provides a theoretical basis and empirical support for designing effective mechanisms to promote regional coordination and balance in TCM service delivery.

## Theoretical framework and research hypotheses

The Uneven Development Theory is an important analytical framework in social sciences, economics, and geography. It is mainly used to explain the phenomenon of unbalanced economic development across regions, countries, or the globe [30]. The theory emphasizes that developed regions with advantageous resource endowments continuously attract high-quality factors such as capital and talent from less-developed regions. As these factors accumulate, developed regions gain stronger regional competitiveness through scale effects. This leads to a further concentration of resources and accelerates the outflow of resources from underdeveloped areas. In contrast, underdeveloped regions experience weakening development capacity and declining competitiveness, resulting in a vicious cycle of continuous resource loss.

In China, the efficiency of medical service delivery in TCM hospitals shows clear regional imbalance. This imbalance is mainly caused by two factors. First, in terms of economic input, developed regions invest more public funding, expand healthcare resources, and attract external high-quality resources. This creates a positive cycle of investment and output. Second, in terms of innovation capacity, developed regions rely on high-quality resources to build medical research platforms, attract talent, and strengthen the innovation ecosystem of medical technology. With advantages in both capital and innovation, developed regions have gradually established a leading position in the efficiency of TCM hospital services. Underdeveloped regions, on the other hand, remain stuck in low-efficiency situations. As a result, the efficiency gap in TCM hospital services continues to widen across regions. Based on this theoretical foundation, the following hypotheses are proposed:

H1: There are significant regional disparities in the allocation efficiency of medical resources in TCM hospitals.

H2: Inter-regional differences are the main cause of the imbalance in medical resource allocation in TCM hospitals.

The Theory of Spatial Equity is a key theoretical framework for analyzing the fair distribution of public service resources across geographic spaces. It is built upon the dual dimensions of geographical and social space and proposes an integrated evaluation system that incorporates spatial accessibility, quality adaptability, and social inclusiveness [31]. The theory innovatively emphasizes that the spatial allocation of public resources must coordinate three core elements: service coverage and accessibility, equal quality of service provision, and protection of vulnerable populations. In the context of resource allocation in traditional Chinese medicine (TCM) hospitals, disparities in economic development among neighboring regions often lead to resource substitution and imbalance. Economically developed regions tend to attract healthcare professionals from surrounding underdeveloped areas, resulting in a decline in the medical service capacity of those less-developed regions. Addressing this problem requires the establishment of cross-regional resource compensation mechanisms and policy coordination systems to ensure the fair distribution of public healthcare resources. These measures are crucial to guarantee equal access to quality medical services for populations across different regions.

From the perspective of spatially coordinated regional development, the allocation of medical service resources is a critical component of the overall regional development strategy. First, developed regions attract TCM medical personnel from surrounding areas by offering higher salaries and better benefits. Second, local governments, under the pressure of performance assessments linked to GDP growth, may reduce investment in public healthcare services. This often leads to a structurally imbalanced allocation of public health expenditure. Third, the tendency of patients to vote with their feet by seeking treatment outside their own regions exacerbates the loss of local healthcare demand and leads to reductions in resource input. This cycle of talent outflow, fiscal compression, and patient loss reflects a fundamental spatial mismatch between healthcare resource supply and patients’ cross-regional service demands. Based on this, the following hypothesis is proposed:

H3: Per capita GDP has a negative spatial spillover effect on the allocation efficiency of medical resources in TCM hospitals.

## Data sources and methods

### Data sources

#### Input and output indicators

For input indicators, this study selected the number of traditional Chinese medicine (TCM) hospitals, the number of hospital beds, and the number of health technicians. Output indicators were categorized into desirable and undesirable outcomes. The desirable outputs included the annual number of outpatient visits, the annual number of discharges, and the bed occupancy rate, which reflect the service utilization and operational performance of TCM hospitals. The undesirable output was the population mortality rate, representing negative health outcomes that TCM services aim to minimize. This study selects population mortality as an undesirable output indicator, based on the following considerations. First, although this indicator has limitations due to its broad scope, it is intrinsically linked to the health outcome objectives of traditional Chinese medicine, particularly the TCM philosophy of disease prevention and early intervention. Second, there is a lack of consistent and complete data on mortality from TCM-specific diseases in the current provincial health statistical systems. Given these data constraints, population mortality represents the most systematically comparable and widely available core health outcome indicator at present.

#### Influencing factor indicators

To comprehensively assess the factors affecting resource allocation efficiency, both external and internal dimensions were considered. External factors comprised per capita gross domestic product (GDP), government health expenditure, urbanization rate, aging rate, and the number of graduates from higher medical institutions per 10,000 population (hereafter referred to as number of graduates). These indicators reflect the broader socioeconomic and demographic context. Internal factors included the physician-to-nurse ratio and the proportion of TCM physicians, which represent institutional capacity and the internal structure of the TCM workforce (Table 1).

**Table 1:** Indicators for Evaluating the Efficiency of Health Resource Allocation and Influencing Factors in TCM Hospitals

Variables	Secondary Indicators
Input Indicators	Number of TCM hospitals
	Number of beds in TCM hospitals
	Number of health technicians in TCM hospitals
Desirable Outputs	Annual number of outpatient visits in TCM hospitals
	Annual number of discharges in TCM hospitals
	Bed occupancy rate in TCM hospitals
Undesirable Output	Population mortality rate
External Influencing Factors	Per capita GDP
	Government health expenditure
	Urbanization rate
	Aging rate
	Number of graduates from higher medical institutions per 10,000 population
Internal Influencing Factors	Physician-to-nurse ratio
	Proportion of TCM physicians

### Research methods

**Fig. 1:**
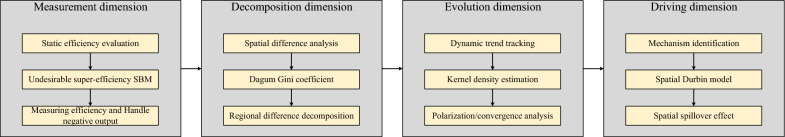
Technical roadmap of resource allocation efficiency research in traditional Chinese medicine hospitals

This study adopts a four-dimensional analytical framework of “Measurement-Decomposition-Evolution-Drivers” (see Fig. 1) to explore the dynamic evolution and underlying mechanisms of resource allocation efficiency in TCM hospitals. First, given the presence of undesirable outputs in TCM medical services, a super-efficiency Slack-Based Measure (SBM) model with undesirable outputs is employed. This model addresses the challenge of ranking multiple efficient decision-making units and incorporating negative output indicators, thus overcoming the limitations of traditional Data Envelopment Analysis (DEA) methods. Second, the Dagum Gini coefficient and its decomposition method are applied to examine regional disparities and their contributions across eastern, central, western, and northeastern China. This spatial analysis effectively addresses the issue of regional balanced development raised in the introduction. Third, kernel density estimation is used to dynamically capture the temporal evolution of TCM hospital efficiency distribution, providing insights into trends of polarization or convergence in resource allocation efficiency. Finally, the Spatial Durbin Model (SDM) is employed to identify key factors influencing the efficiency of TCM hospital services. This approach allows for the analysis of spatial spillover effects between independent and dependent variables.

#### Undesirable-super efficiency SBM model

Traditional DEA models are limited in their ability to account for slack in inputs and outputs, often leading to biased efficiency evaluations. To address this issue, Tone (2001) introduced the SBM model, a non-radial DEA approach that incorporates slack variables directly into the objective function, thereby improving the accuracy of efficiency assessments [30]. Later, in 2022, Tone proposed the Super Efficiency SBM model to further resolve the issue of incomparability among efficient decision-making units (DMUs) [31]. Considering the influence of undesirable outputs, this study adopts a Super Efficiency SBM model with undesirable outputs to measure the efficiency of the evaluated TCM hospitals. The model expression is as follows:

1 $$\begin{gathered} \rho^{*} = min\frac{{\frac{1}{m}\mathop \sum \nolimits_{t = 1}^{m} \left( {\frac{{ \hat{x}}}{{x_{tk} }}} \right)}}{{\frac{1}{{r_{1} + r_{2} }} \times \left( {\mathop \sum \nolimits_{s = 1}^{{r_{1} }} \overline{y}^{d} /y_{sk}^{d} + \mathop \sum \nolimits_{q = 1}^{{r_{2} }} \overline{y}^{u} /y_{qk}^{u} } \right)}} \hfill \\ \overline{x} \ge \mathop \sum \limits_{j = 1, \ne k}^{n} x_{ij} \lambda_{j} ,i = 1,2, \cdots ,m \hfill \\ \overline{y}^{d} \le \mathop \sum \limits_{j = 1, \ne k}^{n} y_{sj}^{d} \lambda_{j} ,s = 1,2, \cdots ,r_{1} \hfill \\ \overline{y}^{u} \ge \mathop \sum \limits_{j = 1, \ne k}^{n} y_{q}^{u} \lambda_{j} ,q = 1,2, \cdots ,r_{2} \hfill \\ \lambda_{j} \ge 0,j = 1,2, \cdots ,n \hfill \\ \hat{x} \ge x_{ik} ,j = 1,2, \cdots ,m \hfill \\ \overline{y}^{d} \le y_{sk}^{d} ,s = 1,2, \cdots ,r_{1} \hfill \\ \overline{y}^{u} \le y_{q}^{u} ,u = 1,2, \cdots ,r_{2} \hfill \\ \end{gathered}$$

In the formula, *r*_1_ and *r*_2_ represent the desirable and undesirable outputs, respectively, while *n* denotes the number of decision-making units (DMUs). The efficiency value, represented by ${\rho }^{*}$, is the result of the measurement, where ${\rho }^{*}$<1 indicates that the DMU is inefficient, and ${\rho }^{*}$≥1 indicates that the DMU is efficient. The measured value is positively correlated with the efficiency value.

#### Dagum Gini coefficient decomposition method

Decomposition methods are commonly used to measure regional differences, including the Theil index, coefficient of variation, and Gini coefficient; However, these methods are not able to further decompose regional differences and compare the distribution of subsamples. The Dagum Gini coefficient can break the above constraints more effectively by decomposing a region into subregions and calculating the total difference, the difference within a region, the difference between regions, and the high variation density. The specific equation of the Dagum Gini coefficient is as follows: In this study, the Dagum Gini coefficient decomposition method was used to study the differential contribution of regions [32].

The overall Gini coefficient *G* value was positively correlated with the overall difference of medical and health resource allocation efficiency of TCM hospitals, the *G*_*w*_ value was positively correlated with the regional differences in the allocation efficiency of medical and health resources allocation of TCM hospitals, the *G*_*nb*_ value was positively correlated with the regional differences in the allocation efficiency of medical and health resources of TCM hospitals, and the *G*_*t*_ value was positively correlated with the impact of different regions on the allocation efficiency of medical and health resources of TCM hospitals.

2 $$G = \frac{{\mathop \sum \nolimits_{j = 1}^{k} \mathop \sum \nolimits_{h = 1}^{k} \mathop \sum \nolimits_{i = 1}^{{n_{j} }} \mathop \sum \nolimits_{r = 1}^{{n_{h} }} \left| {y_{ji} - y_{hr} } \right|}}{{2n^{2} \overline{Y}}}$$

*G* is the overall Gini coefficient, and the larger the value, the greater the difference; *k* is the number of regions; *n*_*j*_ and *n*_*h*_ are the number of provinces in regions *j* and *h*, respectively; y_*ji*_ and y_*hr*_ are the levels of medical and health resource allocation efficiency of TCM hospitals in regions *j* and *h*, respectively. $\overline{Y }$ is the average value of the allocation efficiency of medical and health resources in traditional Chinese medicine hospitals across the country. Before decomposing the Dagum Gini coefficient, the mean value of the allocation efficiency of medical and health resources of TCM hospitals in each province was ranked:

3 $${\text{Y}}_{{1}} \le {\text{Y}}_{{2}} \le ... \le {\text{Y}}_{{\text{k}}}$$

The overall Gini coefficient *G* can be decomposed into regional differential contribution *G*_*w*_, inter-regional differential contribution *G*_*nb*_ and supervariable density contribution *G*_*t*_.

4 $$G = G_{w} + G_{nb} + G_{t}$$

5 $$Gw = \sum\nolimits_{j = 1}^{k} {G_{jj} p_{j} s_{j} }$$

where,${G}_{jj}=\frac{\frac{1}{2{Y}_{j}}\sum_{i=1}^{{n}_{j}} \sum_{r=1}^{{n}_{j}} [{y}_{ji}-{y}_{jr}]}{{n}_{j}^{2}}$, represents the Gini coefficient in region *j*, and *y*_*jr*_ represents the efficiency level of medical and health resource allocation in TCM hospitals in province *r* in region *j*, *p*_*j*_ = *n*_*j*_*/n*, represents the number of provinces in region *j* and the proportion of *n*_*j*_ in the sample size *n*, S_*j*_ = *n*_*j*_ $\overline{Y }$_*j*_*/n* $\overline{Y }$,Where $\overline{Y }$_*j*_ is the overall level of medical and health resource allocation efficiency in* j* region

6 $$G_{nb} = \sum\limits_{j = 2}^{k} {\sum\limits_{h = 1}^{j - 1} {G_{jh} } } \left( {p_{j} s_{h} + p_{h} s_{j} } \right)D_{jh}$$

where,${G}_{jh}=\frac{\sum_{i=1}^{{n}_{j}} \sum_{i=1}^{{n}_{h}} |{y}_{ji}-{y}_{hr}|}{{n}_{j}{n}_{h}\left({Y}_{j}+{Y}_{h}\right)}$,represents the Gini coefficient between region *j* and region *h*, $\overline{Y }$_*j*_is the overall level of medical security in region *j*, and $\overline{Y }$_*h*_ represents the overall level of medical and health resource allocation efficiency in region *h*. *p*_*j*_ = *n*_*j*_*/n*, representing the proportion of the number of provinces in region *j n*_*j*_ to the sample size *n*,${s}_{h}$=$\frac{{\overline{Y} }_{h}{n}_{h}}{n\overline{Y} },{p}_{h}$=$\frac{{n}_{h}}{\overline{n} }$ represents the proportion of the number of provinces in region *h n*_*h*_ to the sample size *n*; *D*_*jh*_ represents the relative influence between region *j* and region *h*, and the calculation formula is *D*_*jh*_ = $\frac{{d}_{jh}-{p}_{jh}}{{d}_{jh}+{p}_{jh}}$, and *d*_*jh*_ represents the difference between the efficiency level of medical and health resource allocation between *j* and *h*, and the calculation formula is: $d_{jh} = \int\limits_{0}^{\infty } {dF_{j} \left( y \right)} \int\limits_{0}^{y} {\left( {y - x} \right)\,dF_{j} \left( x \right)} ,\,p_{jh}$ is the over-variable first-order moment, and the calculation formula is $p_{jh} = \int\limits_{0}^{\infty } {dF_{j} \left( y \right)} \int\limits_{0}^{y} {\left( {y - x} \right)\,dF_{j} \left( x \right)} ,\,\,F_{j}$ is the cumulative density distribution function of the allocation efficiency of medical and health resources in region *j*.

7 $$G_{t} = \sum\nolimits_{j = 2}^{k} {\sum\nolimits_{h = 1}^{j - 1} {G_{jh} \left( {p_{j} s_{h} + p_{h} s_{j} } \right)} } \left( {1 - D_{jh} } \right)$$

The parameters are explained as above.

#### Kernel density estimation method

Kernel density estimation method is an important non-parametric estimation method, which uses a smooth peak function to fit the sample data, and a continuous density curve to describe the distribution of random variables (1) distribution position; (2) shape; (3) ductility; and (4) polarization of the curve [33]. It is generally assumed that the density function of the random variable *X* is:

8 $$f\left( x \right) = \frac{1}{Nh}\sum\limits_{i = 1}^{N} {K\left( {\frac{{X_{i} - x}}{h}} \right)}$$

where *K (·)* is the kernel density function, *n* is the number of observations, and *X*_*i*_ is the independent and identically distributed observations; *x* is the mean value of the observed value, and *h* is the bandwidth. This paper empirically studies the dynamic evolution of the efficiency level of medical and health resource allocation in Chinese medicine hospitals across the country by using Gaussian kernel function [34]. The Gaussian kernel function formula is as follows:

9 $$K\left( x \right) = \frac{1}{{\sqrt {2\pi } }}exp\left( {\frac{ - x}{2}} \right)$$

#### Spatial autocorrelation

**Spatial weight matrix.** The spatial weight matrix based on economic relevance has been widely used in spatial econometrics. This study builds the economic distance weight matrix based on Geographic Economics and spatial econometrics theory. The specific model is as follows

10 $$W\, = \,\left\{ \begin{gathered} \frac{1}{{\left| {\overline{GDP}_{i} - \overline{GDP}_{j} } \right|}},\,\,\,\,\,\,\,\,\,\,i \ne j \hfill \\ 0,\,\,\,\,\,\,\,\,\,\,\,\,\,\,\,\,\,\,\,\,\,\,\,\,\,\,\,\,\,\,\,\,\,\,\,\,\,\,\,\,i = j\,\,\,\,\,\,\,\,\,\,\,\,\,\,\,\, \hfill \\ \end{gathered} \right.$$

where W represents the weight matrix, *GDP*_*i*_ and *GDP*_*j*_ represent the per capita GDP of province *i* and province *j*. In the matrix, the smaller the gap between *GDP*_*i*_ and *GDP*_*j*_, the larger the *W* value, indicating that region *i* and region *j* are closer in economic space.

Moran’s Index (*Moran’s I*) is used to evaluate the overall spatial connection or difference degree of medical and health resource allocation efficiency of traditional Chinese medicine hospitals in the same distribution area. The value range is generally − 1 ~ 1. *Moran’s I* > 0 indicates that there is a positive spatial correlation in the research object. The larger the value, the more obvious the spatial correlation is; *Moran’s I* < 0 indicates that the research object has a negative spatial correlation. The smaller the value, the weaker the spatial correlation and the greater the difference; *Moran’s I* = 0 indicates that there is no spatial correlation among the research objects.

11 $$Moran^{\prime}sI = \frac{{n\sum\nolimits_{i = n}^{n} {\sum\nolimits_{j = 1}^{n} {w\left( {x_{n} - \overline{x} } \right)} } }}{{\sum\nolimits_{i = 1}^{n} {\sum\nolimits_{j = 1}^{n} {w\sum\nolimits_{i = 1}^{n} {\left( {x_{i} - \overline{x} } \right)^{2} } } } }}$$

**Spatial Durbin model.** After Hausman, Wald and LR statistical tests, the P values are significant at 1% confidence level, SDM will not degenerate into SAR or ser, and the goodness of fit and maximum likelihood estimation of the double fixed effect model are the best. The spatial Durbin model is as follows:

12 $$Y_{it} = \rho \mathop \sum \limits_{j = 1}^{n} W_{ij} Y_{jt} + \beta X_{it} + \mathop \sum \limits_{j = 1}^{n} W_{ij} X_{jt} \gamma + \mu_{i} + \delta_{t} + \varepsilon_{it}$$

where,${Y}_{it}$ is the dependent variable, representing the resource allocation efficiency of province *i* in year *t*, and ${Y}_{jt}$ represents the resource allocation efficiency of adjacent province *j* in year *t*; $\rho$ is the spatial lag coefficient of the explained variable, representing the trend and intensity of the spillover effect of the resource allocation efficiency of the province on adjacent provinces; $\gamma$ is the spatial effect coefficient;${\mu }_{i}$*,* ${\delta }_{t}$ and ${\varepsilon }_{it}$ represent fixed effects and random errors in space and time in turn. The measured results of variance expansion factor VIF between independent variables are less than 3, and the model does not have multicollinearity (Table 2).

**Table 2:** Model selection test results

Inspection method	Result
Wald_spatial_lag	177.568***
LR_spatial_lag	178.461***
Wald_spatial_error	134.225***
LR_spatial_error	116.387***
Hausman	54.267***

### Data sources

The data of each province in this article are from the 2016–2022 China Statistical Yearbook, China Health Statistical Yearbook, and national statistical extract of TCM.

## Result

### Measurement and analysis of the efficiency of health resource allocation in traditional Chinese medicine (TCM) hospitals

**Fig. 2:**
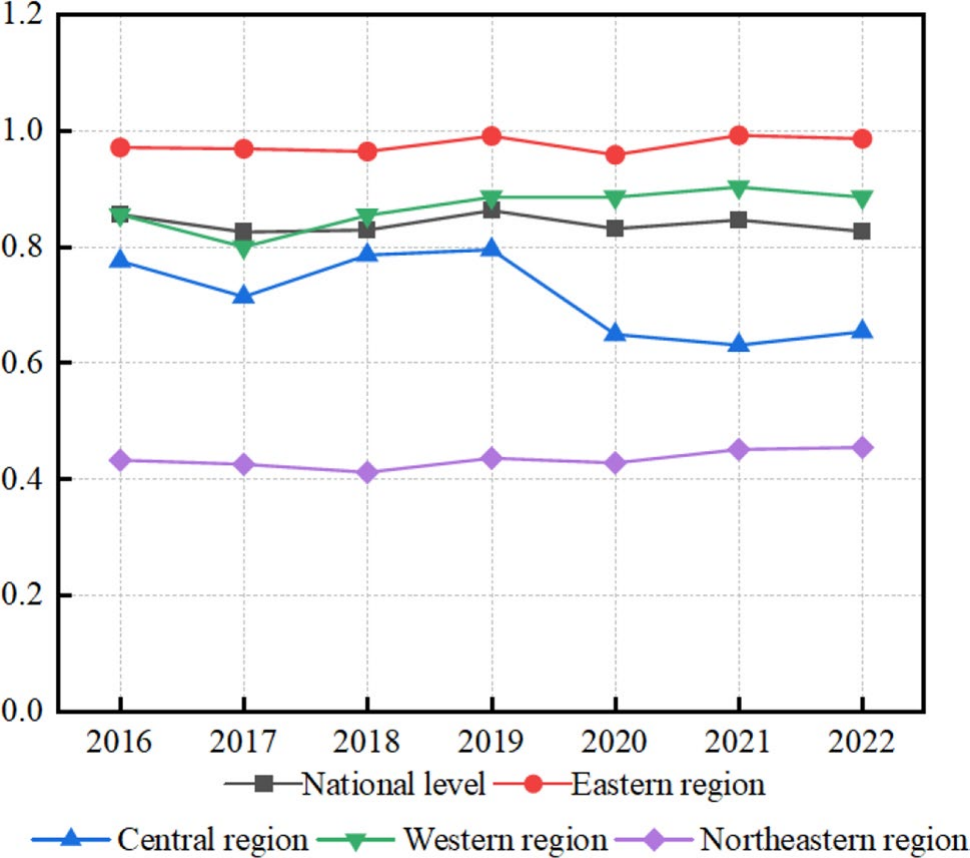
Trends in the efficiency of health resource allocation at the national and four regional levels in China

Analysis of Fig. 2 and Table 3 indicates that, between 2016 and 2019, the efficiency of health resource allocation in TCM hospitals in China showed a steady improvement. This trend was primarily attributed to supportive policy interventions and the diffusion of medical technologies. However, from 2020 to 2022, efficiency levels declined due to the combined impact of the COVID-19 pandemic and medical insurance cost-containment policies, with an average annual decrease of 1.2%.

**Table 3:** Efficiency of health resource allocation in traditional Chinese medicine hospitals across provinces in China

Province	Region	2016	2017	2018	2019	2020	2021	2022	Mean
Beijing	Eastern	1.052	1.031	1.019	1.021	1.027	1.025	1.034	1.031
Tianjin	Eastern	0.694	0.711	0.769	0.752	0.764	0.788	0.725	0.752
Hebei	Eastern	0.668	0.725	0.711	0.694	0.652	0.693	0.649	0.701
Shanghai	Eastern	1.417	1.429	1.469	1.577	1.582	1.615	1.685	1.514
Jiangsu	Eastern	1.022	1.023	1.014	1.112	1.017	1.011	1.025	0.936
Zhejiang	Eastern	1.026	1.072	1.035	1.075	1.104	1.067	1.084	1.062
Fujian	Eastern	0.783	0.712	0.774	0.685	0.746	0.798	0.741	0.746
Shandong	Eastern	0.918	0.759	0.772	0.877	0.893	0.786	0.796	0.855
Guangdong	Eastern	1.088	1.117	1.072	1.124	1.087	1.096	1.115	1.107
Hainan	Eastern	1.039	1.107	1.012	0.991	0.984	1.045	1.012	1.028
Shanxi	Central	0.359	0.371	0.419	0.387	0.362	0.371	0.383	0.371
Anhui	Central	1.018	1.025	0.973	0.998	0.104	0.112	0.107	0.704
Jiangxi	Central	0.802	0.661	0.679	0.758	0.877	0.84	0.891	0.841
Henan	Central	0.763	0.779	0.891	0.874	0.917	0.892	0.921	0.826
Hubei	Central	0.834	0.862	0.857	0.886	0.842	0.891	0.911	0.897
Hunan	Central	0.875	0.752	0.896	0.871	0.799	0.684	0.712	0.845
Liaoning	Northeast	0.487	0.492	0.461	0.473	0.455	0.412	0.398	0.458
Jilin	Northeast	0.376	0.358	0.391	0.416	0.394	0.462	0.474	0.419
Heilongjiang	Northeast	0.436	0.43	0.387	0.422	0.439	0.481	0.494	0.446
Guangxi	Western	0.947	0.922	0.782	0.871	0.776	1.015	0.954	0.911
Chongqing	Western	0.841	0.876	0.712	0.782	0.884	0.783	0.816	0.824
Sichuan	Western	1.062	1.081	1.075	1.077	1.012	1.087	1.045	1.065
Guizhou	Western	0.954	0.178	1.015	1.061	1.091	1.086	1.075	0.932
Yunnan	Western	0.867	0.951	0.924	0.961	1.045	0.984	1.004	0.959
Shaanxi	Western	0.518	0.693	0.635	0.677	0.591	0.641	0.593	0.609
Gansu	Western	0.933	0.986	1.025	1.069	1.087	1.106	1.025	1.014
Qinghai	Western	0.976	0.894	1.042	1.026	1.034	1.027	1.007	0.935
Ningxia	Western	1.138	1.127	1.138	1.097	1.022	1.127	1.091	1.107
Xinjiang	Western	0.791	0.662	0.641	0.728	0.784	0.681	0.716	0.763
Inner Mongolia	Western	0.387	0.436	0.415	0.398	0.412	0.389	0.421	0.413
National average	—	0.835	0.807	0.833	0.858	0.826	0.833	0.830	0.835

At the regional level, the average efficiency exhibited a gradient distribution, characterised by higher levels in the eastern region, relative stability in the western region, and lagging performance in the northeastern region (East > West > Central > Northeast). However, the growth rate of efficiency followed a different pattern, with the northeastern region leading, the western region catching up, the eastern region slowing down, and the central region remaining relatively stagnant (Northeast > West > East > Central). This suggests that regions with lower initial efficiency levels have achieved notable catch-up effects through policy support and late-developer advantages, leading to a significant reduction in absolute regional disparities.

At the provincial level, among the 30 provinces (including municipalities) included in the sample, 17 had average efficiency scores above the national mean of 0.839. Among them, eight provinces and municipalities—including Shanghai (1.514), Ningxia (1.107), and Gansu (1.014)—recorded average efficiency scores exceeding 1.0, forming a cluster of high-efficiency performers. Conversely, five provinces, such as Liaoning (0.458) and Jilin (0.419), had average efficiency scores below 0.5, indicating the presence of low-efficiency areas. It is noteworthy that although Gansu and Ningxia rank among the bottom 30% of provinces in terms of economic development, both achieved high efficiency rankings. This performance can be attributed to their unique resource endowments, such as the production of *Angelica sinensis* in Min County and *Lycium barbarum* in Zhongning, which together account for approximately 45% of the national output. In addition, their relatively low-competition healthcare service environments contributed to higher efficiency, highlighting the compensatory role of resource endowment and cultural tradition in improving health resource allocation.

The findings also indicate that high-efficiency provinces are more likely to face constraints related to diminishing returns to scale. In contrast, provinces with relatively low efficiency have achieved rapid improvements through increased fiscal transfers from the central government and the development of primary healthcare networks, thereby contributing to the gradual convergence of regional differences in allocation efficiency.

A further examination of the outlier results in Table 3 reveals that the efficiency score of TCM hospitals in Anhui Province dropped sharply from 0.998 in 2020 to 0.104. To investigate this anomaly, the study compares regression results based on the full sample from 2016 to 2022 with those from a pre-pandemic adjusted sample covering 2016 to 2019. This comparison aims to explore the underlying reasons for the sharp decline in service efficiency in Anhui's TCM hospitals in 2020.

As shown in Table 4, after controlling for individual and time fixed effects, the coefficient for Anhui Province in the full sample regression is only 0.012, indicating a negligible effect. However, when data from the pandemic period (2020–2022) are excluded, the coefficient rises significantly to 0.376, accompanied by increased statistical significance. These findings indicate that the abnormal and substantial efficiency decline observed in 2020 was primarily driven by the COVID-19 pandemic. The results highlight the severe negative impact of the pandemic on the operational efficiency of TCM hospitals in Anhui Province.

**Table 4:** Comparison of Regression Results on Medical Service Efficiency of TCM Hospitals in Anhui Province

Variable	Full Sample (2016–2022)	Adjusted Sample (2016–2019)
Medical Service Efficiency	0.012**	0.376***
	(0.046)	(0.297)
Control Variables	Controlled	Controlled
Time Fixed Effects	Controlled	Controlled
Individual Fixed Effects	Controlled	Controlled
*R*^2^	0.388	0.579

^***^,**,*Represent significance levels of 1%,5%,and 10%,respectively

### Spatial disparities in the efficiency of health resource allocation in TCM hospitals and their sources

At the national level, the overall disparity in the efficiency of health resource allocation in TCM hospitals in China between 2016 and 2022 experienced two distinct phases: slight fluctuations followed by a gradual upward trend. Specifically, the Gini coefficient increased from 0.166 in 2016 to 0.192 in 2017, decreased to 0.168 in 2019, and then rose again to 0.197 by 2022. This pattern reflects an overall increase in disparity, with greater amplitude of fluctuations observed over time.

In terms of intra-regional differences, the central region exhibited the largest average internal disparity, with a mean Gini coefficient of 0.179, followed by the western (0.139) and eastern (0.127) regions. The northeastern region demonstrated the smallest internal variation. The trend analysis shows that both the eastern and central regions experienced a fluctuating upward trend in intra-regional disparities, suggesting widening gaps in efficiency levels among TCM hospitals within these regions and highlighting growing internal imbalances. In contrast, the western and northeastern regions displayed a clear downward trend in internal disparities, indicating an improvement in the internal equity of resource allocation efficiency.

Regarding inter-regional disparities, the Gini coefficients across the four major regions increased overall during the study period. The ranking of inter-regional mean differences was as follows: Central–West (0.384), West–Northeast (0.341), Central–Northeast (0.304), East–Central (0.195), East–Northeast (0.185), and East–West (0.142). Notably, the top three coefficients exceeded 0.3, indicating substantial efficiency imbalances between regions.

The decomposition of disparities further revealed that the contribution rates of intra-regional, inter-regional, and transvariation density differences remained relatively stable over time. On average, inter-regional disparities contributed the most to overall inequality in resource allocation efficiency, accounting for 53.45% of the total variance. Transvariation density differences contributed 23.37%, while intra-regional disparities contributed the least, at 23.16%. These results suggest that inter-regional differences are the primary source of the overall disparity in efficiency (See Table 5).

**Table 5:** Dagum Gini coefficients and decomposition of contribution rates

Year	Overall Gini coefficient	Contribution rate (%)		
		Within-region	Between-region	Transvariation density
2016	0.166	23.017%	52.916%	24.067%
2017	0.192	24.392%	50.342%	25.265%
2018	0.169	23.311%	51.447%	25.241%
2019	0.168	23.551%	51.496%	24.952%
2020	0.194	22.392%	57.029%	20.578%
2021	0.197	22.426%	56.562%	21.011%
2022	0.197	23.099%	54.392%	22.508%

Year	Within-region disparity				Between-region disparity					
	Eastern	Central	Western	Northeast	East–Central	East–West	East–Northeast	Central–West	Central–Northeast	West–Northeast
2016	0.115	0.131	0.131	0.057	0.155	0.135	0.143	0.383	0.303	0.335
2017	0.125	0.146	0.183	0.07	0.173	0.171	0.179	0.388	0.286	0.344
2018	0.114	0.122	0.144	0.039	0.147	0.137	0.148	0.400	0.315	0.351
2019	0.136	0.12	0.127	0.029	0.158	0.138	0.141	0.388	0.304	0.345
2020	0.131	0.241	0.127	0.031	0.239	0.135	0.222	0.393	0.326	0.350
2021	0.127	0.247	0.136	0.034	0.248	0.138	0.242	0.374	0.295	0.341
2022	0.146	0.247	0.125	0.047	0.247	0.144	0.222	0.368	0.305	0.327

### Dynamic evolution of health resource allocation efficiency in traditional Chinese medicine hospitals

**Fig. 3:**
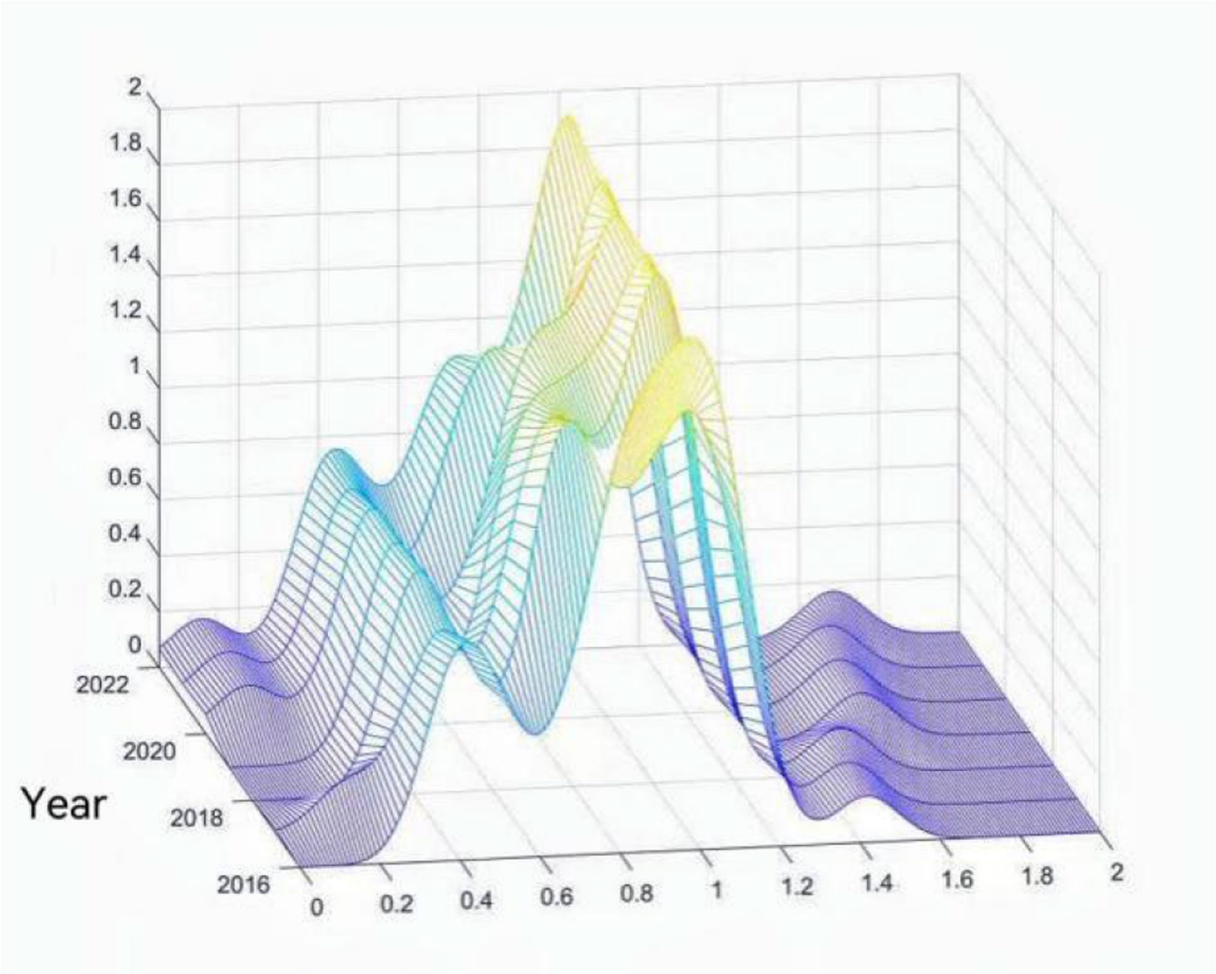
Estimate of Kernel Density of Health Resource Allocation Efficiency in Traditional Chinese Medicine Hospitals

The dynamic evolution trend of health resource allocation efficiency in Traditional Chinese Medicine (TCM) hospitals is illustrated in Figs. 3. Firstly, regarding distribution location, the kernel density curve shifts toward the right, indicating an overall upward trend in the efficiency of health resource allocation in TCM hospitals. Secondly, in terms of distribution shape, the main peak height initially decreases and then increases, while the peak width first expands and then narrows. This suggests that the absolute disparities in resource allocation efficiency across TCM hospitals initially widened but have gradually been controlled and reduced. Thirdly, the distribution exhibits a right-skewed tail that shows signs of convergence, reflecting a considerable gap between the national average efficiency and the presence of a certain number of TCM hospitals with relatively high development levels. Finally, regarding polarization trends, the appearance of a dominant main peak alongside multiple secondary peaks indicates a multi-tiered differentiation pattern in resource allocation efficiency among TCM hospitals, with coexistence of clusters at both low and high efficiency levels.

### Analysis of factors influencing the efficiency of health resource allocation in traditional Chinese medicine hospitals

#### Test for spatial autocorrelation

Table 6 reports the results of the spatial autocorrelation test using Moran’s I. The global Moran’s I values for the efficiency of health resource allocation in Traditional Chinese Medicine (TCM) hospitals are consistently positive and statistically significant at the 1% level, indicating a clear global spatial autocorrelation in efficiency distribution across regions. This spatial clustering pattern justifies the application of a Spatial Durbin Model (SDM) to further examine the determinants of resource allocation efficiency in TCM hospitals.

**Table 6:** Results of Spatial Autocorrelation Test

Year	Moran’s I index	Z-value
2016	0.234***	3.589
2017	0.215***	4.255
2018	0.161***	5.628
2019	0.177***	4.685
2020	0.135***	4.784
2021	0.187***	3.337
2022	0.192***	4.786

^***^,**,*Represent significance levels of 1%, 5% and 10%,respectively

#### Analysis of regression results

Table 7 shows that the spatial autocorrelation coefficient is 0.126, which is statistically significant at the 1% level. This indicates a significant endogenous spatial interaction effect in the efficiency of health resource allocation in TCM hospitals across regions, exhibiting a positive spatial spillover effect.

**Table 7:** SDM Estimation Results

Variable	Direct Effect		Spatial Effect	
	Coefficient	Z-value	Coefficient	Z-value
Per capita GDP	0.146***	0.964	− 0.195**	− 1.276
Government health expenditure	− 0.047	-0.412	0.058	0.475
Urbanization	0.016	2.267	0.027**	3.184
Population density	0.344***	2.439	1.224**	2.775
Aging rate	0.371*	1.775	0.417**	1.985
Number of graduates	0.025***	3.035	0.068**	4.337
Physician-to-nurse ratio	0.133	1.375	− 0.168	-0.561
Proportion of TCM physicians	0.037	1.051	0.283**	1.225

^***^,**,*Represent significance levels of 1,5,and 10%,respectively

Regarding the direct effects of influencing factors, the number of graduates from higher medical education institutions per 10,000 population, the aging rate, and per capita GDP all have a positive direct impact on the efficiency of health resource allocation in TCM hospitals, whereas population density has a direct negative effect on efficiency.

For spatial interaction effects, the number of graduates from higher medical education institutions per 10,000 population, the proportion of TCM physicians, urbanization rate, aging rate, and population density show significant positive spatial spillover effects. In contrast, per capita GDP exhibits a significant negative spatial spillover effect. All these effects are statistically significant, indicating that changes in influencing factors in neighboring regions have spatial spillover effects on the efficiency changes in the local region.

#### Decomposition of spatial effects

To precisely explore the impact of various influencing factors on the efficiency of medical and health resource allocation in TCM hospitals, the spatial effects of the Spatial Durbin Model were further decomposed. Table 8 shows the efficiency of medical and health resource allocation in TCM hospitals is mainly influenced by per capita GDP, urbanization level, aging rate, population density, and the number of graduates from higher medical institutions. The direct effects are significantly positive. Specifically, for every 5% increase in per capita GDP, population density, and the number of graduates per 10,000 people, the efficiency of medical and health resource allocation in TCM hospitals in the corresponding region increases by 13.7%, 29.4%, and 2.2%, respectively. For every 1% increase in urbanization, the efficiency increases by 1.9%. Population density and the proportion of TCM physicians exhibit positive spatial spillover effects on efficiency, whereas per capita GDP exhibits a negative spatial spillover effect.

**Table 8:** SDM Spatial Effects Decomposition Results

Variable	Direct Effect		Spatial Effect		Total Effect	
	Coefficient	Z-value	Coefficient	Z-value	Coefficient	Z-value
Per capita GDP	0.137**	0.863	− 0.086**	− 0.227	0.051	0.242
Government health expenditure	− 0.049	− 0.541	0.066	0.368	0.017	0.683
Urbanization	0.019***	2.024	− 0.007	− 1.671	0.012	0.197
Population density	0.294**	2.752	2.621***	2.398	2.915***	3.221
Aging rate	0.354	1.552	1.472	3.125	1.826	2.778
Number of graduates	0.022**	2.659	0.017	1.984	0.039**	2.662
Doctor nurse ratio	0.157	1.251	− 0.264	− 2.442	− 0.107*	− 2.234
Physician-to-nurse ratio	0.157	1.251	− 0.264	− 2.442	− 0.107*	− 2.234

#### Robustness test

To verify the robustness of the empirical results, the geographic adjacency weight matrix was used to replace the economic distance weight matrix for robustness testing. As shown in Table 9, after replacing the weight matrix, the regression results are similar to the baseline results. The spatial autoregressive coefficient is 0.079 and is significant at the 10% level. The coefficients of the main effects, spatial effects, and the decomposed direct and spillover effects of variables remain largely consistent with the previous analysis, and their significance levels are also basically unchanged. This indicates that the conclusions of the study are robust.

**Table 9:** Robustness test results

Variable	Main effect		Spatial Effect		Direct Effect		Indirect effect		Total Effect	
	Coefficient	Z-value	Coefficient	Z-value	Coefficient	Z-value	Coefficient	Z-value	Coefficient	Z-value
Per capita GDP	0.122**	0.863	− 0.144**	− 1.655	0.256***	0.724	− 0.145	− 0.245	0.111	0.242
Government health expenditure	− 0.038	− 0.228	− 0.068	− 0.773	− 0.022	− 0.488	− 0.074	− 0.854	− 0.096	− 0.422
Urbanization	0.125	1.785	0.066*	2.024	0.037	1.992	− 0.058	− 1.533	− 0.422	− 0.101
Population density	0.238***	3.257	1.521**	− 2.773	0.384**	− 2.375	2.113***	3.651	2.497	3.514
Aging rate	0.354*	2.661	0.362	1.656	0.177	(1.060)	0.981*	2.079	1.158	2.755
Number of graduates	0.077**	2.059	0.125*	3.672	0.224	(2.054)	− 0.125*	− 1.681	0.099	1.063
Doctor nurse ratio	0.265	1.118	− 0.177	− 0.677	0.212	1.782	− 0.388	− 2.337	− 0.176	− 1.422
Physician-to-nurse ratio	0.013	(1.085)	0.397**	1.044	− 0.159	0.875	0.466*	1.951	0.307	0.973

## Discussion

### Steady improvement in efficiency of medical and health resource allocation in TCM hospitals and narrowing regional differences

Despite improvements in recent years, significant regional imbalances persist in the efficiency of healthcare resource allocation among TCM hospitals. Encouragingly, these disparities are gradually transitioning from pronounced imbalance toward a more equitable distribution. This trend reflects the varying endowments of different regions in terms of economic development, population size, policy environment, and medical infrastructure. Economically developed eastern provinces such as Shanghai and Jiangsu have achieved relatively high levels of allocation efficiency, largely due to robust fiscal investment and comprehensive talent recruitment strategies. In contrast, central and western provinces—such as Gansu (Longxi), Jiangxi (Zhangshu), and Anhui (Bozhou), which are major production areas for traditional medicinal materials—have leveraged abundant herbal resources and rich TCM heritage to develop distinctive and locally adapted models of service delivery. However, northeastern regions have lagged behind in efficiency, primarily due to inadequate policy support and the sustained outflow of skilled personnel. Overall, the heterogeneity in resource allocation efficiency reflects the diverse socio-economic and institutional contexts of different regions. Moreover, interregional disparities account for the majority of the observed variation, indicating that the principal driver of inefficiency is not intra-regional differences but rather systemic differences across regions.

To address structural imbalances in the allocation of TCM resources across regions, we recommend establishing a dynamic response mechanism for regional disparities. This mechanism should quantify interprovincial gaps in resource distribution through a three-dimensional assessment framework incorporating economic development level, aging rate, and disease spectrum. Provinces with a Gini coefficient greater than 0.4 and fewer than 1.8 licensed TCM physicians per 10000 population should be prioritized in resource redistribution strategies.

Second, an efficiency-oriented talent supply system should be developed, with full-cycle management encompassing recruitment, training, retention, and utilization. In provinces experiencing significant talent outflow—particularly in the Northwest and Northeast—targeted incentive policies are essential. These should include exemptions from housing settlement fees, personal income tax, and initial employment evaluations, alongside subsidies for housing and research start-up funding, aiming to enhance the attractiveness and stability of local TCM human resources. Third, resource circulation within TCM medical consortiums should be enhanced by adopting a vertical integration and resource sharing model. This includes the regular deployment of urban medical experts to grassroots institutions, the shared use of high-value medical equipment, and the integration and co-analysis of medical records. Such measures are expected to promote more balanced service capacity across urban and rural areas.

Fourth, a national repository of best practices for efficiency improvement should be established to identify and disseminate successful grassroots innovations. For example, Qingyang District TCM Hospital in Chengdu, Sichuan Province, has implemented chain-based management of community TCM clinics, standardized procurement of medical equipment to reduce costs, and improved workforce deployment through mentorship programs. These models provide valuable templates for broader adoption. Finally, a continuous monitoring and improvement system should be instituted to track institutional performance. County-level TCM hospitals that consistently rank at the bottom in efficiency evaluations over multiple years should be subject to a trustee restructuring process, whereby their operations are taken over by provincial-level tertiary hospitals. This top-down approach may catalyze systemic reform and enhance institutional accountability.

### Moderation of multi-polarization in efficiency of medical and health resource allocation in TCM hospitals

The overall efficiency of healthcare resource allocation in TCM hospitals in China has shown a consistent upward trend, accompanied by a gradual mitigation of regional polarization. With sustained economic growth and evolving social conditions, the public’s demand for health services—particularly those rooted in traditional medicine—has significantly increased. This shift has placed greater expectations on TCM hospitals to deliver sufficient and high-quality medical resources aligned with population needs. In response, the Chinese government has introduced a series of supportive policies and legislative measures in recent years, aimed at strengthening the top-level design of the TCM sector and improving the healthcare policy environment. These developments have played a pivotal role in enhancing the allocation efficiency of TCM medical and health resources. However, challenges remain. A stratified pattern of efficiency continues to persist, marked by the coexistence of both high-efficiency and low-efficiency clusters. This phenomenon is primarily driven by the uneven spatial distribution of TCM resources, with high-quality services disproportionately concentrated in economically developed regions. Such imbalances exacerbate interregional disparities in access and efficiency.

To address the current issues of uneven distribution of TCM medical resources and disparities in service efficiency, and taking into account the clinical experience and specialty characteristics emphasized by TCM hospitals, three key strategies are proposed: First, establish a specialty collaboration mechanism by having the provincial TCM administration lead the formation of a TCM hospital specialty alliance. This would involve setting up a specialty alliance construction fund, building a remote consultation platform, and implementing a renowned physician studio inheritance program. The focus should be on supporting the standardized construction of specialty departments in TCM hospitals located in central, western, and northeastern regions, thereby promoting the cross-regional flow and balanced allocation of high-quality TCM medical resources. Second, promote the precise downward extension of appropriate TCM technologies, with an emphasis on the dissemination of three categories of techniques: characteristic TCM diagnostics and treatments, traditional Chinese medicine decoction preparation, and health preservation methods. This ensures effective transmission of classification-based technical guidance, standardized technology promotion, and guarantees for therapeutic efficacy. Third, develop a TCM-specific evaluation system that focuses on creating data standards integrating the four diagnostic methods of TCM—inspection, listening and smelling, inquiry, and pulse-taking—as well as a monitoring system for TCM-specific indicators. This system would accurately identify weaknesses in the medical service capabilities of regional TCM hospitals and enable dynamic assessment of service quality. Based on the evaluation results, precise corrective actions should be taken to address issues such as resource allocation imbalances and insufficient technological application. Utilizing differentiated support and targeted resource allocation will ensure that evaluation outcomes effectively translate into balanced regional resource distribution and improvements in service efficiency.

### Joint influence of internal and external environmental factors on efficiency improvement of medical and health resource allocation in TCM hospitals

The efficiency of healthcare resource allocation in TCM hospitals is shaped by a complex interplay of internal institutional factors and external environmental conditions. Among the most influential external drivers, economic development stands out. Increases in per capita GDP are positively associated with improvements in resource allocation efficiency. Economically advanced regions are better positioned to enhance service delivery capacity through increased fiscal investment and more effective talent aggregation mechanisms. However, regional disparities in income distribution have led to significant inefficiencies. The Gini coefficient between the central and western regions reached a critical threshold of 0.4 in 2018 [35], while that between the western and northeastern regions remained elevated within the range of 0.327–0.351. These figures highlight the pronounced imbalance in resource allocation across regions. This is primarily due to a stronger tendency among high-income groups to seek cross-regional care, which in turn reinforces the siphoning of high-quality resources toward urban centers. Consequently, healthcare accessibility for lower-income populations diminishes. Urbanization also presents a double-edged effect. Initially, rising urbanization rates facilitate resource concentration and improve allocation efficiency. However, beyond a certain threshold, the centripetal pull of urban services intensifies the urban—rural gradient, leading to diminishing returns and exacerbated spatial inequality.

Population aging is another key determinant. For each 1% increase in the proportion of residents aged 65 and above, utilization of TCM services at the primary care level increases by 0.9%. This trend highlights the urgent need for the development of geriatric-appropriate diagnostic and treatment standards within TCM practice. Population density contributes to efficiency gains by reducing administrative oversight costs. In denser regions, more efficient public management systems—such as performance-based budgeting—can be leveraged to optimize the allocation structure of fiscal health expenditures. A critical internal constraint lies in the composition of the TCM workforce. In regions where certified TCM physicians account for less than 18% of the medical workforce, the risk of resource allocation inefficiency increases by 42%. Addressing this structural imbalance calls for the development of an integrated, multi-dimensional training system encompassing academic institutions, traditional mentorship (master-apprentice) programs, and continuing professional education.

Based on the key factors influencing TCM hospital resource allocation efficiency, the proposed policy recommendations offer a focused and practical framework to improve equity and effectiveness nationwide. Strengthening economic foundations in central-western and northeastern regions by aligning TCM fiscal inputs with local GDP growth and promoting region-specific TCM industries can drive sustainable development and resource availability. Introducing income-stratified precision allocation mechanisms ensures that low-income populations receive comprehensive TCM coverage through public financing, while curbing unnecessary high-income patient migration via tiered referral discounts, thereby reducing resource misallocation. Adapting resource distribution to urbanization gradients allows high-urbanization areas to develop specialized regional centers, mid-level urban regions to implement grid-based medical alliances, and low-urbanization counties to provide full basic TCM care, effectively balancing urban–rural disparities. Advancing structural fiscal reforms by setting minimum public health spending quotas for TCM and establishing provincial development funds supports infrastructure modernization and talent cultivation, with differentiated support based on regional needs. Finally, designing aging-responsive allocation schemes by upgrading services for the elderly, and adjusting rehabilitation capacity according to demographic trends addresses the growing demand for geriatric TCM services.

## Conclusion

The efficiency of healthcare resource allocation in TCM hospitals in China has shown a steady improvement overall. However, significant regional disparities persist, underscoring the need for coordinated, multidimensional policy interventions tailored to local conditions. Resource distribution remains uneven, with economically developed regions exhibiting a concentration of high-quality resources, while less efficient areas continue to face challenges. To address this imbalance, enhancing the mobility of resources between urban and rural areas is essential. Differentiated resource allocation plans should be developed specifically for regions where the Gini coefficient exceeds 0.35. Strategies such as talent exchange, technical support, and equipment sharing can be employed to reduce regional disparities. Additionally, establishing and improving supporting systems—such as cross-regional medical insurance settlement, telemedicine, and data sharing—will further facilitate balanced resource distribution. The creation of regional medical consortia can also promote the equitable allocation of healthcare resources across areas.

Several internal and external environmental factors exert substantial influence on the efficiency of TCM healthcare resource allocation. Economic growth, urbanization, population aging, and workforce composition all play critical roles. Notably, increases in per capita GDP and urbanization facilitate resource agglomeration, thereby enhancing efficiency. The integration of aging population policies with resource decentralization strategies has been shown to improve primary care utilization among older adults. Nonetheless, persistent income inequality and imbalances in talent allocation remain major barriers to optimizing resource efficiency across TCM hospitals nationwide.

In response, targeted policy actions are warranted. Strengthening the economic foundation in underdeveloped regions is crucial, including establishing dynamic linkages between regional economic growth and TCM fiscal investment. Implementing income-stratified health insurance schemes that prioritize financial support for economically disadvantaged areas will help address equity concerns. Resource allocation should be aligned with urbanization levels: high-urbanization areas require the establishment of regional TCM medical centers; medium-urbanization regions benefit from grid-based medical alliances; and low-urbanization counties should achieve full coverage for common disease treatment at the county hospital level. Additionally, clearly defining the proportion of public health funding dedicated to TCM and creating specialized provincial funds for infrastructure and talent development will support sustainable growth. Upgrading TCM hospital services to meet the needs of an aging population is imperative, including the creation of integrated geriatric care units and adjustment of rehabilitation capacity based on demographic aging indices. Collectively, these measures will promote a more balanced, efficient, and equitable allocation of TCM healthcare resources nationwide.

Although disease-specific mortality rates related to TCM hospital services are theoretically more targeted indicators, current data limitations prevent effective quantification and statistical analysis. As a result, population mortality remains the most systematically comparable core health outcome indicator available, highlighting a key challenge that future modernization of TCM evaluation systems must address. We will continue to monitor the development trends and cutting-edge research in TCM medical services, continuously refining and optimizing our research methods and indicator systems to more accurately reflect the progress in TCM service efficiency.

## Data Availability

No datasets were generated or analysed during the current study.

## Abbreviations

TCM: Traditional Chinese medicine
GDP: Gross domestic product
DEA: Data envelopment analysis
SBM: Super-efficiency slack-based measure
SDM: Spatial Durbin model
